# Supplementary material for: Identification of photoperiod-induced specific miRNAs in the adrenal glands of Sunite sheep (Ovis aries)
Source: Front Vet Sci. 2022 Jul 22;9:888207. doi: 10.3389/fvets.2022.888207 (PMC9354845; doi:10.3389/fvets.2022.888207)
Supplement: Supplementary file 1 [file Data_Sheet_1.ZIP › Supplementary file/miRDeep2 file.zip!/miRDeep2 file/Know_Structure/SP42A2/oar-mir-1197.pdf]

miRBase precursor : oar-mir-1197
Total read count : 92
oar-miR-1197-5p read count : 1
oar-miR-1197-3p read count : 82
remaining reads : 9

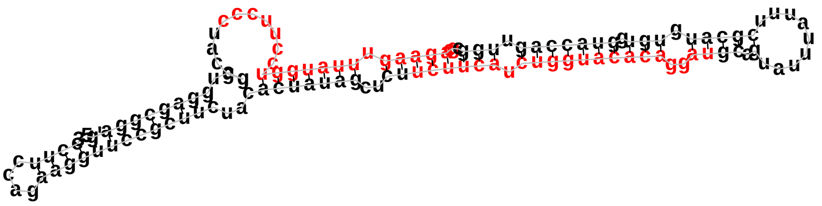

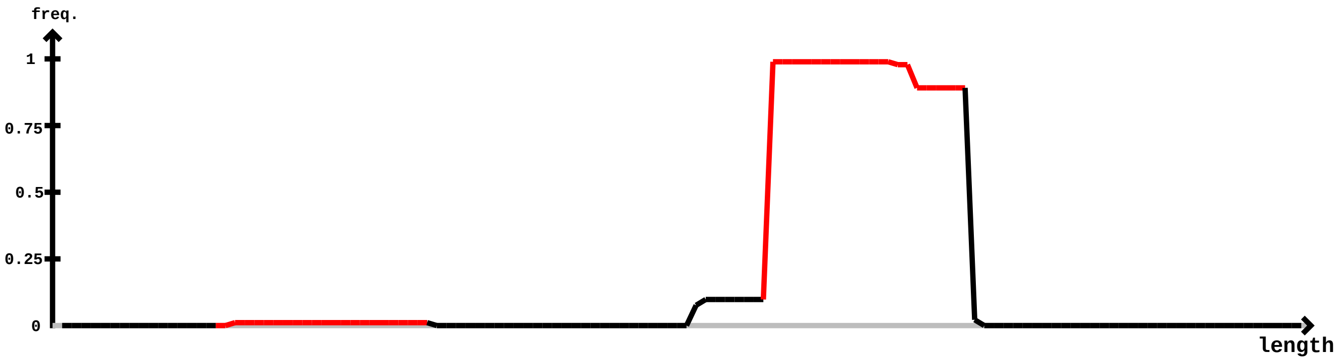


```
                                                                               oar-miR-1197-3p
                 oar-miR-1197-5p
5'- gaggcgaggucggcaucccuuccugguauuugaagacgcgguugaccaugguguguacgcuuuauuuaugacguaggacacauggucuacuucuucucgauaucacaucuucgccuuggaagaccuucc  -3'   exp
    (((((((((..(...........(((((((.(((((...(((.(((((((.(((.(((((.........).))))..))))))))))).))))))))..))))))))..)))))))))((((....))))        reads   mm
    .................ccuuccugguauuugaagacg...........................................................................................        1       0
    .................................................................uuaugacguaggacacauggu...........................................        1       0
    .................................................................uuaugacguaggacacauggucu.........................................        6       0
    ..................................................................uaugacguaggacacauggucu.........................................        2       0
    .........................................................................uaggacacauggucuacuucu...................................        80      0
    .........................................................................uaggacacauggucuacuucuu..................................        2       0
```
